# Autologous Bone Marrow-Derived Mesenchymal Stem Cells Modulate Molecular Markers of Inflammation in Dogs with Cruciate Ligament Rupture

**DOI:** 10.1371/journal.pone.0159095

**Published:** 2016-08-30

**Authors:** Peter Muir, Eric C. Hans, Molly Racette, Nicola Volstad, Susannah J. Sample, Caitlin Heaton, Gerianne Holzman, Susan L. Schaefer, Debra D. Bloom, Jason A. Bleedorn, Zhengling Hao, Ermias Amene, M. Suresh, Peiman Hematti

**Affiliations:** 1 Comparative Orthopaedic Research Laboratory, School of Veterinary Medicine, University of Wisconsin-Madison, Madison, Wisconsin, 53706, United States of America; 2 Department of Comparative Biosciences, School of Veterinary Medicine, University of Wisconsin-Madison, Madison, Wisconsin, 53706, United States of America; 3 UW Veterinary Care Hospital, School of Veterinary Medicine, University of Wisconsin-Madison, Madison, Wisconsin, United States of America; 4 Department of Medicine, School of Medicine & Public Health, University of Wisconsin-Madison, Madison, Wisconsin, 53705, United States of America; 5 Department of Medical Sciences, School of Veterinary Medicine, University of Wisconsin-Madison, Madison, Wisconsin, 53706, United States of America; 6 Department of Pathobiological Sciences, School of Veterinary Medicine, University of Wisconsin-Madison, Madison, Wisconsin, 53706, United States of America; 7 University of Wisconsin Carbone Cancer Center, Madison, Wisconsin, 53705, United States of America; INEB, PORTUGAL

## Abstract

Mid-substance rupture of the canine cranial cruciate ligament rupture (CR) and associated stifle osteoarthritis (OA) is an important veterinary health problem. CR causes stifle joint instability and contralateral CR often develops. The dog is an important model for human anterior cruciate ligament (ACL) rupture, where rupture of graft repair or the contralateral ACL is also common. This suggests that both genetic and environmental factors may increase ligament rupture risk. We investigated use of bone marrow-derived mesenchymal stem cells (BM-MSCs) to reduce systemic and stifle joint inflammatory responses in dogs with CR. Twelve dogs with unilateral CR and contralateral stable partial CR were enrolled prospectively. BM-MSCs were collected during surgical treatment of the unstable CR stifle and culture-expanded. BM-MSCs were subsequently injected at a dose of 2x10^6^ BM-MSCs/kg intravenously and 5x10^6^ BM-MSCs by intra-articular injection of the partial CR stifle. Blood (entry, 4 and 8 weeks) and stifle synovial fluid (entry and 8 weeks) were obtained after BM-MSC injection. No adverse events after BM-MSC treatment were detected. Circulating CD8^+^ T lymphocytes were lower after BM-MSC injection. Serum C-reactive protein (CRP) was decreased at 4 weeks and serum CXCL8 was increased at 8 weeks. Synovial CRP in the complete CR stifle was decreased at 8 weeks. Synovial IFNγ was also lower in both stifles after BM-MSC injection. Synovial/serum CRP ratio at diagnosis in the partial CR stifle was significantly correlated with development of a second CR. Systemic and intra-articular injection of autologous BM-MSCs in dogs with partial CR suppresses systemic and stifle joint inflammation, including CRP concentrations. Intra-articular injection of autologous BM-MSCs had profound effects on the correlation and conditional dependencies of cytokines using causal networks. Such treatment effects could ameliorate risk of a second CR by modifying the stifle joint inflammatory response associated with cranial cruciate ligament matrix degeneration or damage.

## Introduction

Mid-substance rupture of the canine cranial cruciate ligament (CrCL) and associated stifle osteoarthritis (OA) is an important veterinary health problem [1]. Cruciate ligament rupture (CR) causes stifle joint instability and is not typically associated with contact trauma. Contralateral CR often develops, suggesting that the disease is a complex trait in which both genetic and environmental factors may increase risk of CR [1]. The dog is an important model for human anterior cruciate ligament (ACL) rupture, which is particularly common in young athletes [2]. In human beings, rupture of a graft repair or the contralateral ACL is also common [3,4].

Intrinsic healing of the CrCL/ACL is a research focus in veterinary and human medicine. Mesenchymal stem cells (also commonly known as mesenchymal stromal cells, MSCs) are multipotent adult progenitor cells that reside in virtually all tissues, including bone marrow (BM) [5]. These cells can differentiate into mesenchymal lineage cells, such as bone, cartilage, or connective tissue. Consequently, there is great interest in the therapeutic potential of MSCs as a cell-based tissue repair treatment in orthopaedics. Fresh BM mononuclear cells or culture-expanded BM-MSCs can enhance healing of partial CrCL transection in rats [6,7]. Bio-enhanced healing of CrCL autografts for treatment of the unstable stifle using growth factors, such as platelet-derived growth factor, has also been demonstrated in caprine and porcine models [8,9].

The early phase of the CrCL rupture mechanism, in which damage to CrCL matrix leads to partial CR in a stable stifle is not understood. Also, little is known about intrinsic healing of partial CR. In dogs, stifle osteoarthritis (OA) is typically present at CR diagnosis. In affected stifles, lymphocytic-plasmacytic synovitis is present throughout the joint. Inflammatory changes initially develop in the stable stifle [1,10,11] and include disturbances to T lymphocyte subsets within the stifle synovium [12]. CrCL structural properties are significantly reduced by stifle synovitis [13], suggesting that synovitis contributes to development of a mid-substance rupture in the absence of contact trauma [10]. In experimental models, partial CRs lack the capacity for healing and typically progress to CR [14]. In dogs with unilateral CR and a contralateral partial CR, radiographic stifle synovial effusion and osteophytosis is predictive of a second CR [1,15,16]. Synovitis in unstable CR stifles is also predictive for risk of a second CR in dogs [17]. Collectively, these findings suggest that stifle synovitis is a therapeutic target in the early phase of CR in dogs.

MSCs home to damaged tissues and contribute to repair through many mechanisms including paracrine effects via secretion of growth factors, cytokines, anti-fibrotic or angiogenic mediators, and extracellular matrix proteins [18,19]. MSCs also possess immune-regulatory functions both in vitro and in vivo [19,20]. Autologous MSCs have immune-modulatory properties and can act by suppressing many functions of immune cells in an MHC-independent manner. Immunosuppressive behavior is driven by effects on T lymphocyte function [21], dendritic cell maturation, B cell function [22,23], and macrophage phenotype [24] and can influence arthritis severity [25].

Previous work has shown that allogeneic BM-MSCs can enhance healing of partial CR in rodent models [6,7], but the effect of autologous MSC treatment on the inflammatory response that develops within stifle joints affected with partial CR is unclear. The present study was designed to evaluate the effect of systemic and local administration of autologous cultured BM-MSCs on stifle joint inflammation in dogs with partial CR. Our hypothesis was that systemic and local BM-MSC treatment would influence circulating T cell subsets, as well as modulate the inflammatory response both systemically and locally. We identified a suppressive effect of autologous BM-MSCs on serum and synovial CRP and related immune-modulatory effects on serum and stifle synovial fluid cytokines that was sustained over an 8-week period.

## Materials and Methods

### Dogs

Twelve dogs with unilateral complete CR in the index stifle and a stable contralateral stifle with partial CR were prospectively recruited at the UW Veterinary Care Hospital, University of Wisconsin-Madison for a clinical trial between November 2013 and February 2015. Passive stifle stability was assessed with testing for cranial translation of the tibia under sedation [26]. Inclusion criteria were: clinical signs of lameness in the **index** pelvic limb, palpable cranial translation of the tibia relative to the femur indicative of complete CR, no evidence of **contralateral** cranial tibial thrust based on joint palpation under sedation, and radiographic evidence of bilateral stifle synovial effusion or osteophytosis, indicating contralateral partial CR [1,10,16]. Dogs were excluded if history and physical examination suggested traumatic injury or other stifle pathology, such as patellar luxation, if there was a history of previous stifle surgery, or if the contralateral stifle was unstable. Age, weight, gender, and previous history were recorded.

### Ethics statement

All procedures were performed in accordance with the recommendations in the Guide for the Care and Use of Laboratory Animals of the National Institutes of Health and the American Veterinary Medical Association, and with specific approval from the Animal Care & Use committee of the School of Veterinary Medicine, University of Wisconsin-Madison (V1070). Specific informed written consent from owners was also obtained.

### Experimental design

**Fig 1:**
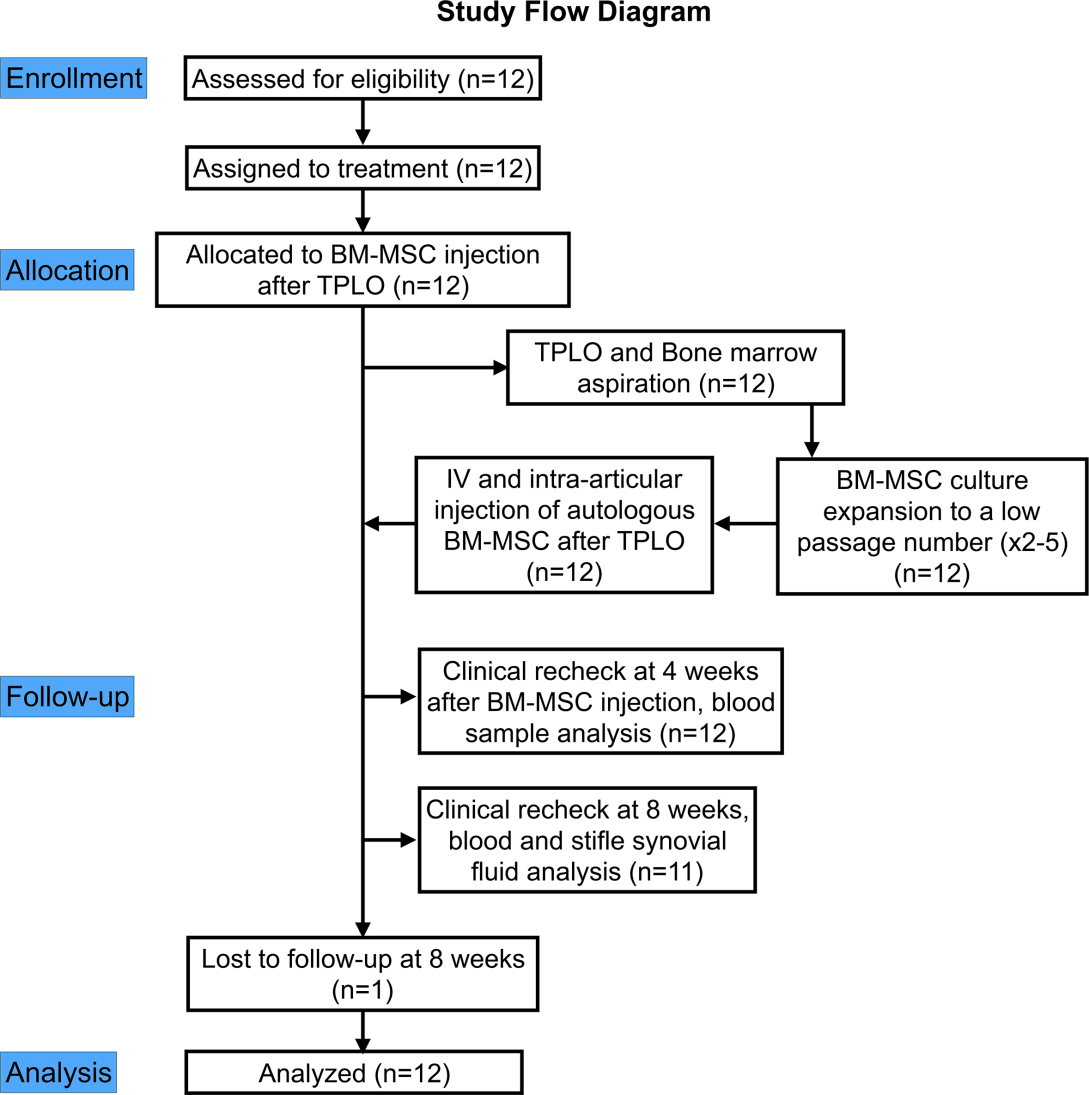
Study design flow diagram illustrating treatment allocation, time line, follow-up, and analysis. TPLO–tibial plateau leveling osteotomy for treatment of stifle instability from CR. BM-MSC–Bone marrow-derived mesenchymal stem cells.

The study design is illustrated in Fig 1. After ex vivo culture expansion of MSCs from an autologous BM aspirate that was collected during tibial plateau leveling osteotomy (TPLO) treatment of the index stifle with CR, BM-MSCs were administered by intravenous injection and intra-articular injection into the contralateral stifle with partial CR. Bilateral stifle radiographs, and blood and bilateral stifle synovial fluid samples were collected at diagnosis and study entry, and 8 weeks after BM-MSC injection. A blood sample was also collected at 4 weeks after BM-MSC injection. Circulating T lymphocyte subsets were analyzed by flow cytometry at each visit after isolation of peripheral blood mononuclear cells (PBMCs). C-reactive protein (CRP) and cytokine concentrations in serum and synovial fluid were determined by ELISA. Total nucleated cell count (TNCC) was also estimated in synovial fluid [27] at diagnosis and at 8 weeks after BM-MSC injection.

### Radiography

Initially, weight-bearing latero-medial stifle radiographs were made bilaterally to determine the functional length of the CrCL under load [28]. Orthogonal cranio-caudal and medio-lateral radiographic views of the stifle were subsequently obtained bilaterally under sedation. Synovial effusion and osteophytosis were used to evaluate the severity of stifle OA [1]. These criteria were selected based on high reproducibility [29]. The most important early radiographic sign of stifle OA in dogs with CR is development of synovial effusion and associated compression of the infrapatellar fat pad [1,16]. Synovial effusion and osteophytosis were graded subjectively on a scale from 0–2 (0—normal, 1—mild, 2—severe) [1]. Both cranial and caudal stifle joint spaces were examined in this assessment. Cranially, the extent of effusion and the shape of the infrapatellar fat pad were considered. Caudal bulging of the joint capsule was also evaluated during grading. Osteophytosis was graded subjectively on a scale from 0–3 (0—normal, 1—mild, 2—moderate, 3—severe) based on the severity of osteophytosis at the margins of the stifle joint [1]. In addition, the tibial plateau angle (TPA) was calculated from the lateral radiographic views of the index and contralateral tibias with the stifle and tarsus held in ninety degrees of flexion. The functional length of the CrCL (mm) (CrCL_d_) was determined from the distance between the femoral and tibial attachments on weight-bearing lateral radiographs [28]. CrCL_d_ was normalized to body size using the length of the patella. Radiographic scoring was performed by a single observer (PM).

### Arthroscopy

Before TPLO treatment, a medial parapatellar mini-arthrotomy was made in the index stifle with CR. The joint was examined using a 2.7 or 2.9 mm 30° rigid arthroscope (Linvatec, Largo, FL) placed in the mini-arthrotomy incision. Stifle joint regions (lateral and medial pouches, lateral and medial femoro-tibial joint compartments, the intercondylar notch, and the femoro-patellar joint) were examined arthroscopically [11]. Severity of synovitis and cruciate ligament damage was graded arthroscopically using a standardized scoring system [11]. Three parameters describing macroscopic inflammation were evaluated for each joint region: Synovial hypertrophy, vascularity, and synovitis [11]. Additionally, global synovitis severity was scored using a visual analog scale (VAS) (0–100) for each stifle joint, with 0 representing no inflammation, and 100 signifying the most severe inflammation [11]. The CrCL and the caudal cruciate ligament (CdCL) were inspected and probed for evidence of pathologic change, including fiber rupture, color change, and hypertrophy of ruptured ends of ligament fascicles. The extent of fiber disruption was estimated with a calibrated arthroscopic probe. Damage to the CrCL and the CdCL was graded as 0 –normal, 1 –superficial fraying, 2 –moderate damage with 10 to 50% fiber rupture, 3 –severe damage with >50% fiber rupture. The lateral and medial menisci were also inspected and probed for tears.

### Preparation of donor MSCs for autologous treatment

During TPLO treatment, femoral BM aspirates were collected using a 13g Jamshidi needle that was placed into the distal femoral metaphysis through the proximal end of the mini-arthrotomy incision. Physiologic saline (20ml 0.9% NaCl containing 20IU/ml of heparin) was injected in the femoral marrow cavity using a syringe and the saline mixed with bone marrow tissue was aspirated back into the syringe. The aspiration procedure was then repeated one more time.

BM aspirates were maintained at room temperature overnight and then processed for culturing the next day. Mononuclear cells were isolated by Leucosep tubes (Greiner Bio-one, Monroe, NC, USA) according to the manufacturer’s protocol. Red blood cells (RBC) were lysed with incubation for 3 minutes in RBC lysis buffer and mononuclear cells were suspended in alpha minimum essential media (α-MEM, Mediatech Inc., Nanassas, VA) supplemented with 10% fetal bovine serum (FBS- Hyclone, Logan, UT, USA), 1X non-essential amino acids (NEAA, Mediatech Inc., Manassas, VA), 4 mM L-glutamine (Mediatech Inc., Manassas, VA), and 100U/mL penicillin, 0.01 mg/mL streptomycin sulfate (Life Technologies, Grand Island. NY). After adherence of stromal cells to plastic plates, the culture media was changed to remove non-adherent cells after 24 hours. BM cells were cultured in a 37° C incubator with 5% CO_2_−95% humidified atmosphere. Media was changed 3 times per week. BM cells were expanded until passage 2 to 5, at which time they were used for autologous treatment *in vivo*.

### Autologous BM-MSCs administration

Systemic and local injections of BM-MSCs were performed as soon as sufficient numbers of cells were available. Plated BM-MSC cells were detached from culture flasks with trypLE (Life Technologies, Grand Island. NY) and counted using a hemocytometer. Dogs received a dose of 2x10^6^ autologous BM-MSCs/kg that were administered intravenously in 0.9% saline at a concentration of 20x10^6^ BM-MSCs/ml. Cell viability at injection was confirmed to be >90% [30]. In addition, 5x10^6^ BM-MSCs were injected into the contralateral stifle joint with partial CR.

### Flow cytometry of BM-MSCs and PBMCs

A BD FACS Calibur flow cytometer (BD Biosciences, San Jose, CA) and FlowJo software (Tree Star Inc., Ashland, OR) were used for these analyses. Fluorochrome-labeled antibodies for anti-canine CD34, CD44, CD45, CD29, and CD90 were used for cell surface marker analysis of cultured bone marrow cells. Cultured cells were fixed in 2% paraformaldehyde in PBS pH 7.4 and stored protected from light at 2–8°C for flow cytometry analysis. Histogram plots were examined for mononuclear cell populations of interest to confirm that cultured cells were BM-MSCs: CD44 (YKIX337.8.7), CD90 (YKIX337.217) and CD29 (TS2/16) positive and CD34 (clone 1H6) and CD45 (clone YKIX716.13) negative (CD44, CD90, CD34, CD45, Abd Serotec, Raleigh, NC; CD29, Biolegend, San Diego, CA) [31].

For analysis of circulating T lymphocyte subsets, PBMCs were isolated using cell separation tubes (BD Vacutainer^TM^ CPT^TM^, Becton Dickinson, Franklin Lakes, NJ) from blood samples. The volume of blood collected was recorded to allow absolute quantification of cell subsets. PBMC were prepared for flow cytometry [12], and stained with fluorochrome-labeled monoclonal antibodies for anti-canine CD3 (clone CA17.2A12), CD4 (clone YK1X302.9), and CD8 (clone YCATE55.9) (Abd Serotec, Raleigh, NC). Dual staining with CD3 was used to confirm the identity of CD4^+^ and CD8^+^ cells lymphocytes, and exclude the possibility of counting CD4^+^ neutrophils, since CD4 is also expressed on canine neutrophils [32]. Cell counting was performed using a hemocytometer. Cells were fixed in 2% paraformaldehyde in PBS pH 7.4 and stored protected from light at 2–8°C before flow cytometry analysis. Scatter plots were examined for T cell populations of interest including CD3^+^CD4^+^, CD3^+^CD8^+^, CD3^+^CD4^-^CD8^-^, and CD3^+^CD4^+^CD8^+^.

### Analysis of synovial TNCC

Direct smears of synovial fluid were stained with Wright-Giemsa stain. Quality control for direct smears was as described [27]. Manual counting of synovial cells on each smear was performed using a validated method for TNCC estimation [27]. All nucleated cells in a field were counted, including those with pyknotic and karyorrhectic nuclei, cells forming groups, and naked nuclei. A cell was considered within the counting field if at least half the cell was within the microscopic field. Nucleated cells in fifteen 400x fields were counted and the mean number of nucleated cells per 400x field was determined. After correction for the dimensions of the 400x field area, TNCC was then estimated using the regression formula (y = 0.45x-0.36) [27].

### Analysis of serum and synovial CRP

CRP is a marker of inflammation that is typically elevated in dogs with inflammatory arthritis and falls in response to anti-inflammatory therapy [33,34]. Serum and synovial CRP was measured using a canine-specific ELISA (ICL Inc., Portland, OR). Synovial fluid/serum CRP ratio was calculated.

### Analysis of serum and synovial cytokines

Serum and synovial fluid cytokine concentrations were measured for a panel of 13 cytokines (Granulocyte macrophage colony-stimulating factor GM-CSF, interferon gamma IFNγ, chemokine [C-X-C motif] ligand 1 CXCL1, CXCL8, CXCL10, chemokine [C-C motif] ligand 2 CCL2, interleukin 2 IL-2, IL-6, IL-7, IL-10, IL-15, IL-18, tumor necrosis factor alpha TNFα), using a canine-specific magnetic bead assay (Milliplex MAP Canine Cytokine Panel, EMD Millipore Corporation, Billerica, MA). The assay was performed according to the manufacturer’s instructions using a Luminex MAGPIX with XPONENT software ver. 4.2 (Luminex, Madison, WI).

### Statistical Analysis

Data were reported as mean±standard error or median (range) as appropriate. The Shapiro-Wilk’s test was used to determine if data approximated a normal distribution. The Wilcoxon signed rank test or the Friedman test was used to determine whether outcome variables were significantly influenced by treatment. The Pearson or Spearman Rank correlation tests were used to determine whether T lymphocyte subsets were related to radiographic severity of synovial effusion or OA on radiographic views made at diagnosis. The Spearman Rank correlation test was used to determine whether dependent variables at diagnosis were correlated with development of a second CR. A one-tail test was used for analysis of radiographic and CRP data, as it has been shown that development of CR is positively correlated with radiographic severity score and elevated CRP values [1,33]. Results were considered significant at *P*<0.05. Correlation and Bayesian network (BN) plots were also drawn using R version 3.1.3 (http://www.r-project.org) to examine the interrelationship and changes in the pattern of the network of inflammatory biomarkers before and after BM-MSC injection [35]. The Markov blanket of the outcome variable (i.e. BM-MSC injection treatment) was identified as the nodes that had strong conditional dependencies with the outcome. The nodes were averaged over 500 networks and a threshold of 0.52 was chosen for the nodes present in the final network. The averaged BN analysis is a data learning method used to construct a graphical model to understand the conditional dependencies among variables represented as nodes in a network [35].

## Results

### Clinical and arthroscopic findings

Summary data are presented in Table 1. There was 1 male, three castrated males, and 7 ovariohysterectomized females. Breeds were Saint Bernard (n = 2), American Pit Bull Terrier (n = 2), mixed breed (n = 2), and one dog of each of the following breeds: Labrador Retriever, Golden Retriever, English Springer Spaniel, Rottweiler, Newfoundland, Mastiff. Left (n = 6) and right (n = 6) complete CR were identified. One dog was lost to follow-up at 8 weeks, although the owner reported that the dog was doing well. In one dog (#12), very mild cranial drawer (Grade II sprain) was present in the partial CR stifle. In the complete CR stifle, complete anatomic tearing of the CrCL was identified in 4 dogs arthroscopically. In the other 8 dogs, remaining CrCL fibers were slack. Some degree of axial fiber tearing was often identified on medial and lateral menisci. Medial meniscus tears were identified in 5 of 12 dogs.

**Table 1:** Clinical findings.

Parameter	Mean±SE or median	Range
Dog age (years)	4.93±0.7	1.6, 9.6
Body weight (kg)	41.8±5.5	16.4, 80.0
4 week recheck (days)	27±1.3	21, 33
8 week recheck (days)	58±0.7	56, 63
Arthroscopic synovitis score	45	32, 53
Arthroscopic VAS synovitis score	57.4±6.0	27, 87
CrCL arthroscopic damage score	3	2, 3
CdCL arthroscopic damage score	1	0, 2

**Abbreviations**: SE, standard error; VAS, visual analogue scale; CrCL, cranial cruciate ligament; CdCL, caudal cruciate ligament.

### MSC production and administration

**Fig 2:**
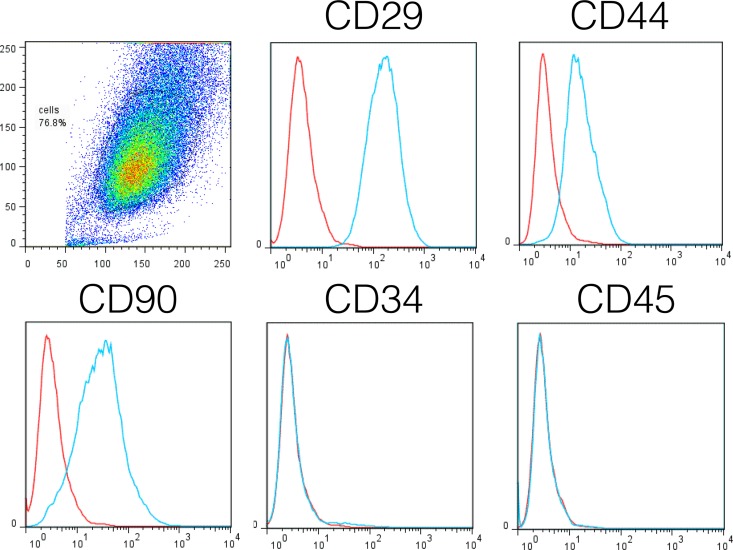
The phenotype of cultured bone marrow-derived mesenchymal stem cells (BM-MSCs) from each dog was confirmed before autologous injection. Expression of CD29, CD34, CD44, CD45, and CD90 by cultured cells was evaluated by flow cytometric analysis using anti-CD29, anti-CD34, anti-CD44, anti-CD45, and anti-CD90 antibodies, after selection of the cell population of interest. Control isotype antibody is plotted in red in each histogram plot. Cultured cells were confirmed as BM-MSCs by their appearance in culture and by the CD29^+^CD44^+^CD90^+^CD34^-^CD45^-^ phenotype.

No complications were detected clinically with BM aspiration or BM-MSC injections. Treatment was performed at passage 2 (n = 7), 3 (n = 2), 4 (n = 1), and 5 (n = 2). The time from BM aspiration to BM-MSC injection was 47±10.3 days (range 19, 139). BM-MSCs were CD29^+^, CD44^+^, CD90^+^, and CD34^-^ and C45^-^, confirming the MSC phenotype (Fig 2). The treatment dose of BM-MSCs was 1.8E6±0.19 cells/kg.

### Radiography

**Fig 3:**
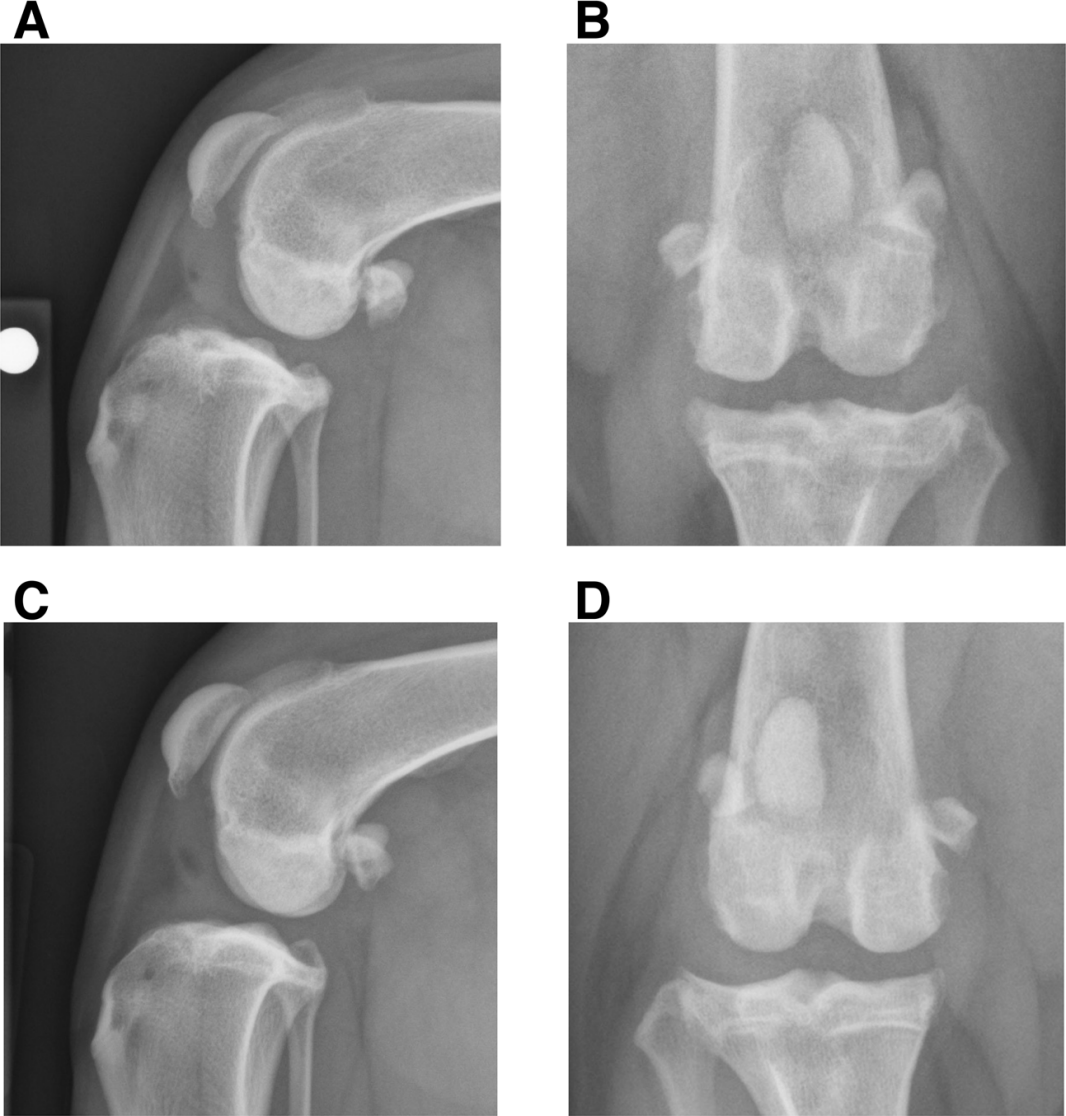
Radiographic synovial effusion and osteophytosis in dogs with unilateral complete cruciate ligament rupture and contralateral partial cruciate ligament rupture. Orthogonal stifle radiographic views of the unstable stifle with complete cruciate ligament rupture (CR) (**A,B**) and the contralateral stable stifle with partial CR (**C,D**) in a four old mixed breed dog at trial entry. Synovial effusion with compression of the infrapatellar fat pat and caudal bulging of the joint capsule is evident in both stifles. Peri-articular osteophytosis is present in both stifles and is particularly evident on the distal pole of the patella, the trochlea ridges of the distal femur, and the margins of the tibial plateau. Osteophytosis is more severe in the stifle with complete CR in this dog. Overall, at diagnosis and trial entry, stifle synovial effusion (*P*<0.05) and osteophytosis (*P* = 0.01) were increased in the unstable stifle with complete cruciate ligament rupture (CR), when compared with the contralateral stable stifle with partial CR. Stifle radiographs were graded subjectively used a standardized scoring method [1].

At diagnosis, synovial effusion (*P*<0.05) and osteophytosis (*P* = 0.01) were increased in the complete CR stifle, compared with the contralateral partial CR stifle (Table 2, Fig 3). There were no significant changes in effusion or osteophytosis after BM-MSC injection. At diagnosis, CrCL_d_ was increased in the complete CR stifle, compared with the contralateral stifle (*P*<0.005). CrCL_d_ was lower in the TPLO-treated stifle at 8 weeks (*P* = 0.06).

**Table 2:** Stifle radiographic parameters before and after injection with bone marrow-derived mesenchymal stem cells.

Radiographic parameter	Partial CR stifle		Complete CR stifle	
	Diagnosis	8 weeks	Diagnosis	8 weeks
Synovial effusion severity score	1.5 (1, 2)	2 (1, 2)	2 (2, 2)^a^	2 (1, 2)
Osteophytosis severity score	1 (1, 3)	1 (1, 3)	2 (1, 3)^b^	2 (2, 3)
CrCL_d_ (mm)	33.5±2.9	32.1±3.3	38.9±2.9^c^	33.7±2.4^d^

**Note:** Instability in the complete CR stifle was treated with tibial plateau leveling osteotomy at diagnosis. CrCL_d_ represents the functional length of the ligament under load as determined by weight-bearing radiography [28].

^a^*P*<0.05

^b^*P*<0.01

^c^*P*<0.005 versus contralateral stifle.

^d^*P* = 0.06 versus complete CR stifle at diagnosis. **Abbreviations**: CR, cruciate ligament rupture; CrCL, cranial cruciate ligament.

### T lymphocyte subsets in the peripheral circulation

Absolute numbers of the CD4^+^, CD8^+^, CD4^-^CD8^-^ and CD4^+^CD8^+^ T lymphocyte subsets in the peripheral circulation at 4 and 8 weeks after BM-MSC treatment were not significantly changed (Table 3). Numbers of circulating CD4^+^ and CD4^-^CD8^-^ T lymphocyte subsets were generally lower at 4 weeks and similar to baseline at 8 weeks. The proportion of CD8^+^ T lymphocytes was lower at 8 weeks, compared with diagnosis (*P* = 0.06). Median counts were lower at 4 and 8 weeks after treatment, when compared with diagnosis. Several individual dogs (#1, #4, #10, #12) with high counts for CD3^+^, CD4^+^, CD8^+^, and CD4^-^CD8^-^ T cell subsets showed suppression of T cell subset numbers at 4 and 8 weeks after BM-MSC treatment (S1 and S2 Tables). Dogs #4 and #10 developed contralateral CR. Other individual dogs (#3, #6, #9) showed elevation in CD3^+^, CD4^+^, CD8^+^, and CD4^-^CD8^-^ T cell subset numbers at 4 and 8 weeks after BM-MSC treatment, when compared with diagnosis (S1 and S2 Tables). Dog #9 also developed contralateral CR. High numbers of circulating and intra-articular T cells may influence joint inflammation and development of CR.

**Table 3:** T lymphocyte subset numbers in the peripheral circulation before and after BM-MSC treatment.

Cell Type		Diagnosis	4 weeks	8 weeks
CD3^+^	Absolute count	0.051 (0.001, 0.357)	0.007 (0.000, 0.898)	0.044 (0.000, 0.172)
CD3^+^	Proportional count	0.81 (0.63, 0.94)	0.765 (0.65, 0.92)	0.80 (0.50, 0.85)
CD4^+^	Absolute count	0.023 (0.001, 0.171)	0.004 (0.000, 0.614)	0.026 (0.000, 0.114)
CD4^+^	Proportional count	0.415 (0.20, 0.59)	0.44 (0.32, 0.66)	0.48 (0.25, 0.53)
CD8^+^	Absolute count	0.011 (0.000, 0.110)	0.002 (0.000, 0.189)	0.008 (0.000, 0.058)
CD8^+^	Proportional count	0.18 (0.10, 0.57)	0.16 (0.10, 0.43)	^a^0.15 (0.09, 0.33)
CD4^-^CD8^-^	Absolute count	0.031 (0.001, 0.099)	0.004 (0.000, 0.378)	0.023 (0.000, 0.069)
CD4^-^CD8^-^	Proportional count	0.365 (0.25, 0.63)	0.375 (0.23, 0.54)	0.38 (0.29, 0.64)
CD4^+^CD8^+^	Absolute count	0.000 (0.000, 0.005)	0.000 (0.000, 0.001)	0.000 (0.000, 0.012)
CD4^+^CD8^+^	Proportional count	0.00 (0.00, 0.00)	0.00 (0.00, 0.14)	0.00 (0.00, 0.07)

**Note**: Absolute counts (E06/ml of blood).

^a^*P* = 0.06 versus diagnosis. **Abbreviations**: BM-MSC, bone marrow-derived mesenchymal stem cells.

### Synovial and serum markers of inflammation

There were no significant changes in synovial fluid TNCC at eight weeks after BM-MSC treatment in either stifle. In partial CR stifles, TNCC was 0.54 (0.00,11.45) and 0.66 (0, 2.87) x1000/μL at diagnosis and 8 weeks after BM-MSC injection respectively. In the CR stifle, TNCC was 0.06 (0.00, 4.30) and 0.84 (0.00, 30.09) x1000/μL at diagnosis and 8 weeks after BM-MSC injection, respectively.

**Fig 4:**
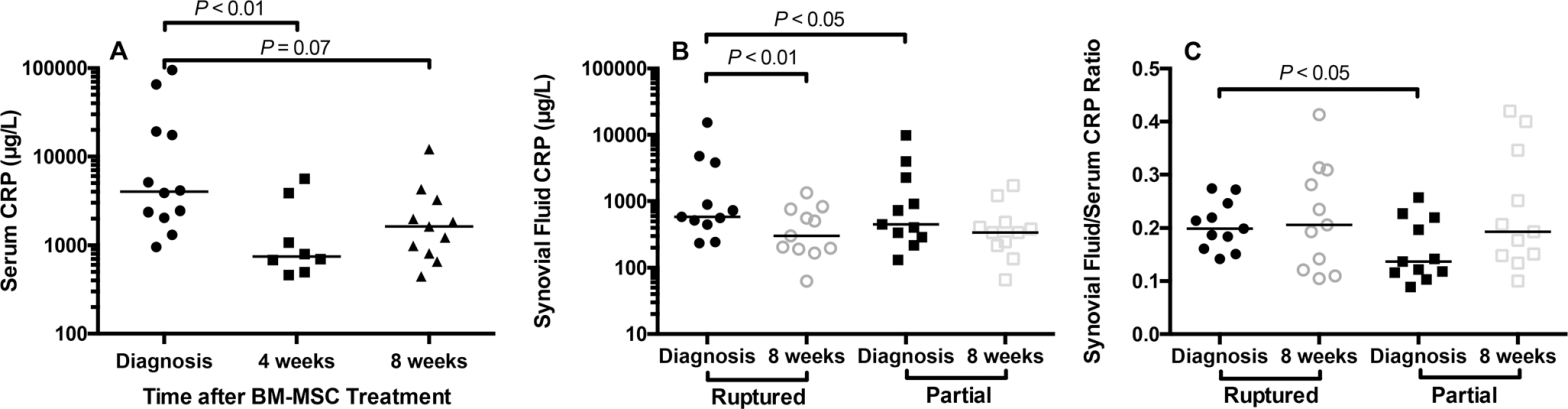
Effect of autologous bone marrow-derived mesenchymal stem cell (BM-MSC) treatment on serum and stifle synovial fluid C-reactive protein (CRP) in dogs with cruciate ligament rupture (CR). (**A**) Median serum CRP concentration was significantly decreased at 4 weeks after MSC treatment, compared with diagnosis. Median serum CRP was also lower at 8 weeks, compared with diagnosis. (**B**) At diagnosis, CRP concentrations in synovial fluid were increased in the unstable stifle with CR (Ruptured), compared with the stable contralateral stifle with partial CR (Partial). In the unstable CR stifle and the stable partial CR stifle, median synovial fluid CRP was lower at 8 weeks, compared with diagnosis. This change was significant for the unstable stifle (*P*<0.05). **(C)** Median synovial fluid/serum CRP ratio was similar at 8 weeks, when compared with diagnosis in the CR stifle. In the partial CR stifle, median synovial/serum CRP ratio was higher at 8 weeks after BM-MSC treatment, compared with diagnosis.

**Fig 5:**
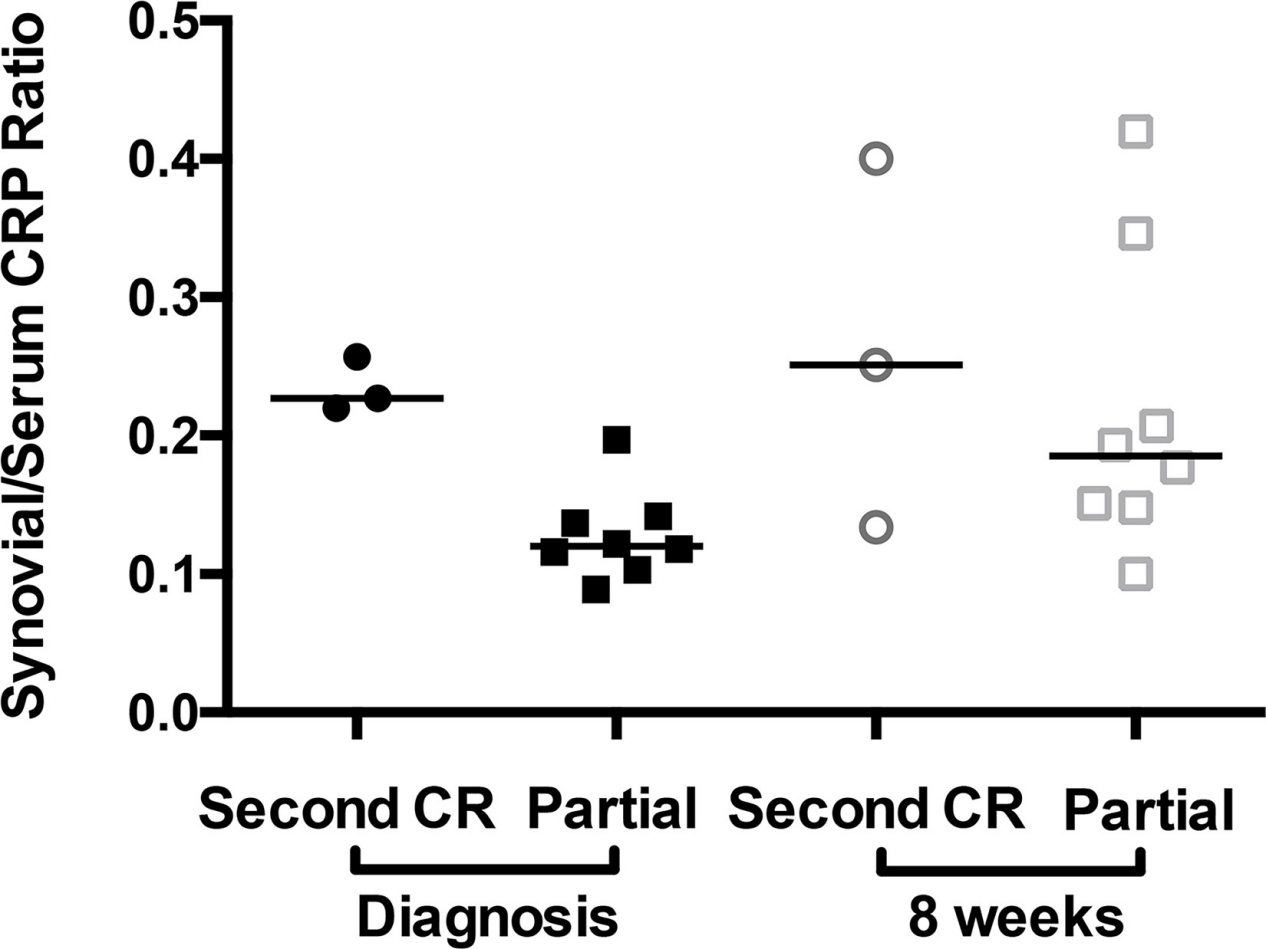
Synovial fluid/serum C-reactive protein (CRP) ratio in stifle joints of dogs with stable partial cruciate ligament rupture (CR) before and after treatment with bone marrow-derived mesenchymal stem cell treatment. At diagnosis, synovial fluid/serum CRP ratio was elevated in the partial CR stifles of dogs that developed a second CR (n = 3) within the study period, when compared with the dogs that did not develop a second CR (n = 8). Synovial CRP was not measured in one dog because of insufficient sample volume. Synovial fluid/serum CRP ratio values were higher at 8 weeks in the dogs that did not experience CR within the study period.

Serum CRP at diagnosis, 4, and 8 weeks after BM-MSC injection was 4015 (955, 94978), 747 (460, 5594), and 1632 (445, 12142) μg/L respectively (Fig 4). Serum CRP was decreased at 4 weeks, compared with diagnosis (*P*<0.01). Synovial CRP in partial CR stifle at diagnosis and 8 weeks after BM-MSC injection was 452 (131, 9815) and 341 (66, 1713) μg/L (Fig 4). Synovial CRP in complete CR stifles at diagnosis and 8 weeks after BM-MSC injection was 586 (235, 15265) and 303 (63, 1338) μg/L, a significant reduction (*P*<0.01). At diagnosis, synovial CRP and synovial/serum CRP ratio in the complete CR stifle were increased, when compared with the contralateral partial CR stifle (*P*<0.05) (Fig 4). Synovial/serum ratio was used to assess synovial inflammation relative to background systemic inflammation. In dogs that developed a second CR, synovial fluid/serum CRP ratio was elevated in the partial CR stifle at diagnosis. At 8 weeks, synovial/serum CRP ratio was higher than at diagnosis in partial CR stifles that did not experience a second CR (Fig 5), suggesting ongoing stifle synovitis.

**Fig 6:**
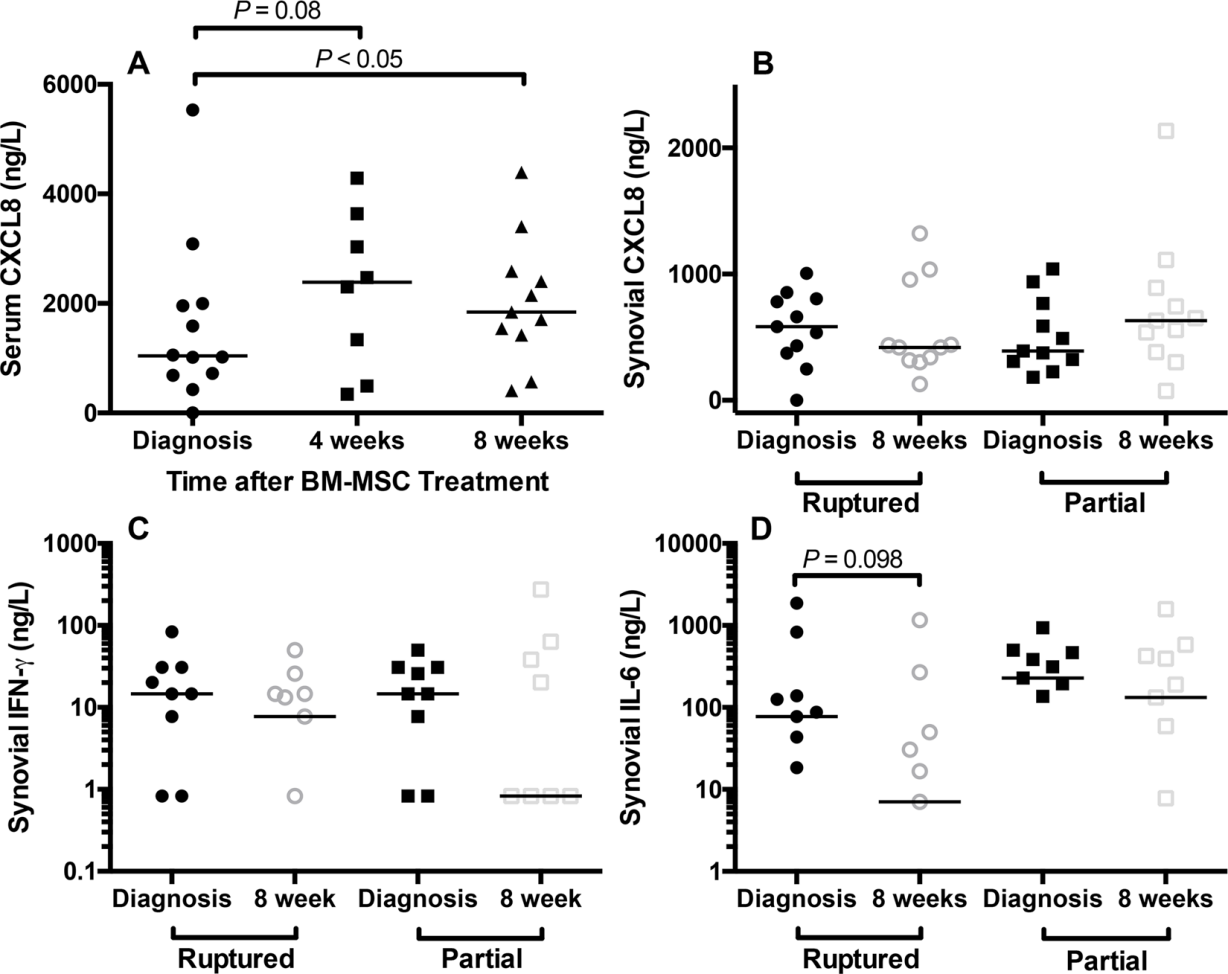
Effect of autologous bone marrow-derived mesenchymal stem cell (BM-MSC) treatment on serum and stifle synovial fluid chemokine [C-X-C motif] ligand 8 (CXCL8) and stifle synovial fluid interferon gamma (IFN-γ) and interleukin 6 (IL-6). (**A**) Median serum CXCL8 concentration was significantly increased at 8 weeks after MSC treatment, compared with diagnosis. Median serum CXCL8 was also higher at 4 weeks, compared with diagnosis. (**B**) Synovial CXCL8 concentrations were similar at diagnosis and at 8 weeks in both stifles. (**C**) Suppression of synovial fluid IFNγ was evident in both stifles after intra-articular and intravenous injection of BM-MSCs, particularly the partial cruciate rupture stifle that was injected with BM-MSCs. Serum IFNγ was not significantly influenced by treatment. (**D**) High concentrations of synovial IL-6 were identified in the stable partial cruciate rupture stifles, when compared with the unstable stifles with cruciate ligament rupture. Median concentrations of synovial IL-6 were lower after BM-MSC treatment in both stifles. As the y axis is logarithmic in C and D, only values greater than zero are plotted.

Serum CXCL8 was increased at 8 weeks 1841 (408, 4392) ng/L, compared with diagnosis 1040 (0, 5534) ng/L (*P*<0.05). Serum CXCL8 was also higher at 4 weeks 2388 (344, 4287) ng/L, when compared with diagnosis (*P* = 0.08) (Fig 6). Synovial CXCL8 was similar at diagnosis and at 8 weeks in both stifles (Fig 6). BM-MSCs are known to secrete CXCL8 [36] and elevations in serum CXCL8 likely reflect BM-MSC injection.

Serum CXCL1, CCL2, GM-CSF, IFNγ, IL-6, IL-10, IL-15, and IL-18 were not significantly altered by BM-MSC injection. Median concentrations were similar at each time point. Serum IFNγ was detected in three dogs at diagnosis (143.5, 42.3, and 261.0 ng/L). IFNγ was not detected in any dogs at 4 weeks and was detected in 2 dogs at 8 weeks. Serum IL-6 was detected in three dogs at diagnosis, 1 dog at 4 weeks and 2 dogs at 8 weeks. Serum IL-10 was detected in 4 dogs at diagnosis (84.7, 12.8, 193, and 124.9 ng/L), but was not detected in any dog at 4 weeks and was detected in 3 dogs at 8 weeks. Median serum IL-18 was lower at 4 (7.4 ng/L) and 8 weeks (7.4 ng/L), when compared with diagnosis (10.7 ng/L), but this difference was not significant. Synovial CXCL1, CCL2, GM-CSF, IL-15, IL-10, and IL-18 were not significantly influenced by BM-MSC injection over the study period and median concentrations were similar at each time point. Suppression of synovial IFNγ from 14.7 (0, 83.8) to 0.8 (0, 274) ng/L was evident after BM-MSC injection in the partial CR stifle (Fig 6). Synovial IL-6 concentrations were higher in the partial CR stifle, compared with the unstable stifle (Fig 6). Similar to the effect on IFNγ, suppression of synovial IL-6 from 87.5 (0, 1871) to 7.05 (0, 1160) ng/L in the complete CR stifle and 193.9 (0, 500) to 132.0 (0, 1582) ng/L in the partial CR stifle was seen with BM-MSC injection (Fig 6).

IL-2 was not detected in serum or synovial fluid from the CR stifle. In the partial CR stifle, IL-2 was detected in 1 dog at both time points and 1 dog at diagnosis. IL-7 was detected in the serum of one dog at diagnosis, 4 and 8 weeks after BM-MSC injection (465, 403, and 408 ng/L, respectively). Synovial IL-7 was detected in both stifles at diagnosis in 1 dog. In two other dogs, synovial IL-7 was detected in the stable stifle with partial CR at 8 weeks. CXCL10 was not detected in serum in any dog at diagnosis. In 1 dog at 4 and 8 weeks, serum CXCL10 was 11.1 and 12.8 ng/L, respectively, and in 1 dog at 8 weeks it was 2.5 ng/L. Synovial CXCL10 was not detected in the complete CR stifle. In the partial CR stifle, synovial CXCL10 was not detected at diagnosis, but was detected in two dogs at 8 weeks. TNFα was not detected in serum. In the complete CR stifle, TNFα was detected in synovial fluid in 1 dog at diagnosis and 1 dog at 8 weeks. In one other dog, it was detected at both time points. In the partial CR stifle, TNFα was not detected at diagnosis, but high concentrations were found in 1 dog at 8 weeks.

In the 3 dogs that developed a second CR, serum CXCL8 was higher at 4 and 8 weeks, compared with diagnosis. Serum CCL2 was higher at 8 weeks, compared with diagnosis in two of these dogs. Serum GM-CSF (82.3 ng/L), IFNγ (284.7 ng/L), IL-10 (89.9 ng/L) were detected at 8 weeks in one of the dogs with a second CR at 82 days after diagnosis, but were not detected at diagnosis or 4 weeks.

**Fig 7:**
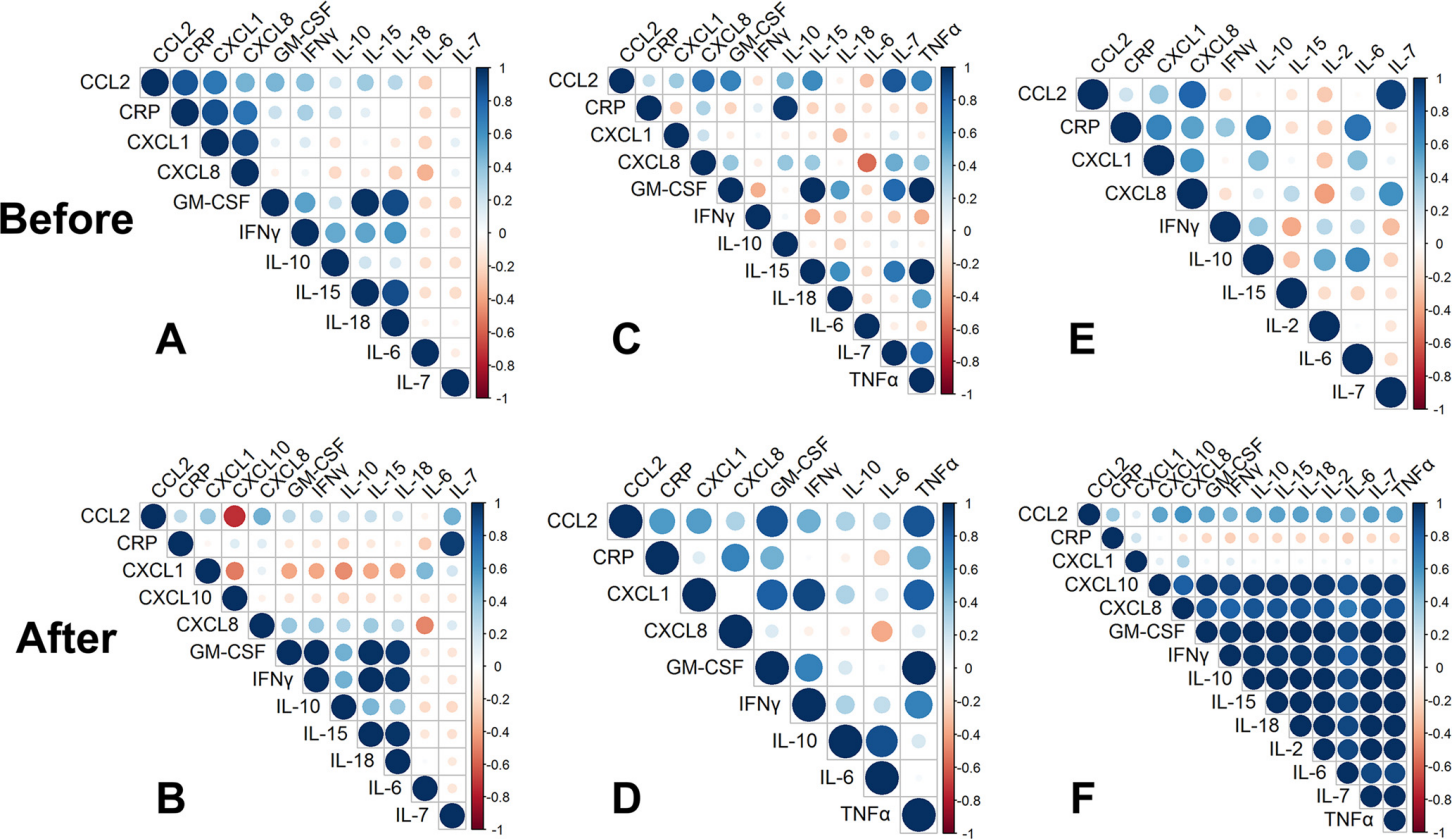
Correlation plots of the panel of inflammatory biomarkers in serum and synovial fluid before and after autologous bone marrow-derived mesenchymal stem cell (BM-MSC) injection. (**A,B**) In serum, strong positive correlations between CXCL1, CXCL8, and CCL2 and CRP became much weaker after BM-MSC injection. The weak correlation between IL-7 and CRP became much stronger after BM-MSC injection. (**C,D**) In synovial fluid from the complete CR stifle, correlation of CCL2 and TNFα with other cytokines was strengthened after BM-MSC injection. The strong correlation between CRP and IL-10 became much weaker after BM-MSC injection. (**EF**) In synovial fluid from the stable partial CR stifle, cytokines in the network were weakly correlated before treatment and these correlations because much stronger after BM-MSC injection, with the exception of CXCL1. Correlations between CRP and IL-6, IL-10, CXCL1 and CXCL8 became much weaker after BM-MSC injection.

**Fig 8:**
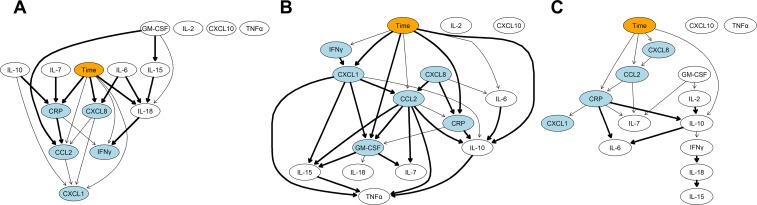
Bayesian networks derived from a panel of inflammatory biomarkers in serum and synovial fluid before and after autologous bone marrow-derived mesenchymal stem cell (BM-MSC) injection. (**A**) In serum, CRP was influenced by IL-7 and IL-10 as well BM-MSC injection. CXCL8 and IL-18 were also influenced by BM-MSC injection and in turn IL-18 influenced IFNγ concentrations. (**B**) In synovial fluid in the complete CR stifle, there was a complex network in which CRP, CXCL1, GM-CSF, and IL-10 showed conditional dependencies with BM-MSC injection. Ultimately, TNFα concentrations were strongly influenced by several pro-inflammatory cytokines. (**C**) In synovial fluid in the stable partial CR stifle, there was a much less complex network in which no strong dependencies on BM-MSC injection were identified. CRP, CCL2, CXCL8 and IL-10 were weakly influenced by BM-MSC injection. The orange node indicates the time-dependent effect of BM-MSC injection. The light blue colored nodes indicate the Markov blankets of the network that identify a subset of cytokines directly influenced by BM-MSC injection. Thicker arcs indicate higher connection strength between nodes (i.e. variables).

Correlation and network analysis of CRP and cytokine concentrations was performed to evaluate changes in the pattern of markers before and after BM-MSC injection. In serum, correlation of inflammatory markers before and after BM-MSC treatment were similar. However, strong positive correlations between CXCL1, CXCL8, and CCL2 and CRP became much weaker after BM-MSC injection. The weak correlation between IL-7 and CRP became much stronger after BM-MSC injection (Fig 7). BN representation of serum cytokine data showed that serum CRP was influenced by IL-7 and IL-10 as well BM-MSC injection. CXCL8 and IL-18 were also influenced by BM-MSC injection and IL-18 in turn influenced IFNγ (Fig 8).

In synovial fluid from the complete CR stifle, correlation of CCL2 and TNFα with other cytokines and of CXCL1 with several other cytokines was strengthened after BM-MSC injection. The strong correlation between CRP and IL-10 became much weaker after BM-MSC injection (Fig 7). Averaged BN analysis identified a complex interaction in which CRP, CXCL1, GM-CSF, and IL-10 were influenced by BM-MSC injection. Ultimately, TNFα concentrations were strongly influenced by several pro-inflammatory cytokines (Fig 8).

In synovial fluid from the partial CR stifle, a number of cytokines were weakly correlated before treatment. These correlations because much stronger after BM-MSC injection with the exception of CXCL1. Correlations between CRP and IL-6, IL-10, CXCL1 and CXCL8 became much weaker after BM-MSC injection (Fig 7). The averaged BN analysis identified a much less complex network in which no strong dependencies on BM-MSC injection were identified. CRP, CCL2, CXCL8, and IL-10 were weakly influenced by BM-MSC injection (Fig 8).

### Development of a second complete cruciate ligament rupture

Three of 11 dogs (27%) developed a second complete CR at 19, 82, and 144 days after diagnosis and trial entry (S3 Table, Figs 4 and 5). Breeds of these dogs were a Newfoundland, an American Pit Bull Terrier, and a Golden Retriever. Severity of synovial effusion (S_R_ = 0.58, *P*<0.09) in the partial CR stifle was correlated with development of a second CR. Synovial/serum CRP ratio at diagnosis in the stable partial CR stifle was also correlated with a second CR (S_R_ = 0.78, *P*<0.01). TNCC in the stable partial CR stifle, synovitis severity in the complete CR stifle assessed arthroscopically, T lymphocyte numbers and proportions in the peripheral circulation, normalized CrCL_d_ in the partial CR stifle, and serum CRP at diagnosis were not correlated with second CR. Serum CCL2 (S_R_ = -0.64, *P*<0.05) and serum IL-18 (S_R_ = -0.54, *P* = 0.095) were inversely correlated with development of a second CR. These data suggest that synovial/serum CRP ratio in stable partial CR stifles is an important predictive marker of risk complete CR.

## Discussion

The study results demonstrate that autologous canine BM-MSC injections can elicit an anti-inflammatory effect over an eight-week period after intravenous and intra-articular injection of the partial CR stifle in dogs with unilateral complete CR and contralateral stable partial CR. CRP concentrations were suppressed in both serum and stifle synovial fluid. Analysis of the synovial/serum CRP ratio at diagnosis in partial CR stifles suggested that this parameter is a predictive biomarker of progressive fiber tearing in the CrCL. In partial CR stifles, higher synovial/serum CRP ratios were found at 8 weeks after BM-MSC treatment, when compared with diagnosis. Additional anti-inflammatory treatment may provide a synergistic effect to more completely block the synovial inflammation associated with progressive CrCL fiber tearing in partial CR stifles. Our results provide evidence that supports use of BM-MSC treatment as an anti-inflammatory treatment for progressive CrCL fiber tearing in dogs with partial CR. In this initial work, we focused effort on studying stifles with partial CR, as a less demanding model than stifles with complete CR, where healing of the CrCL represents a much greater treatment challenge.

The cohort of dogs in this report exhibited typical CR clinical signs [1]. Meniscal damage was commonly detected arthroscopically and 27% developed a second CR. The three dogs that developed a second CR within the study period were from breeds with a relatively high prevalence of CR [37]. In dogs, stifle synovitis develops in the early phase of the condition in association with gradual fiber rupture in the CrCL/CdCL complex [10]. The synovial sheath surrounding the CrCL has a functionally important, but poorly understood, role in CrCL homeostasis that includes a protective barrier function with regard to the surrounding stifle joint synovial fluid [38,39]. Ligament matrix physiological environment is influenced by surrounding synovial fluid because of a blood-ligament barrier in the CrCL microvasculature [39] and the extra-synovial intra-articular location of the CrCL/CdCL complex. In dogs, development of stifle synovitis influences risk of a second CR [17]. This observation likely explains the reduction in CrCL structural properties that follows induction of stifle synovitis experimentally [13].

We found severity of stifle synovitis assessed by synovial CRP and radiographic synovial effusion and osteophytosis was elevated in the CR stifle at diagnosis, when compared with the contralateral partial CR stifle, similar to previous work [11]. The lower CrCL_d_ value after TPLO supports efficacy as a joint stabilizing treatment [40,41]. CRP, which is mainly produced by hepatocytes, may reflect or contribute to molecular mechanisms of disease states [42]. The pro-inflammatory cytokines IL-6 and IL-1 are the main regulators of CRP [42]. We identified high IL-6 in stifle synovial fluid, particularly in the partial CR stifles, and a strong correlation between synovial IL-6 and CRP that was eliminated by BM-MSC injection. Synovial fluid and serum CRP concentrations are correlated in dogs with CrCL rupture [11]. Synovial IL-6 is also correlated with serum CRP, suggesting that serum CRP reflects synovitis in humans with OA [42], although these parameters were only weakly correlated in this report. In humans with OA, serum CRP typically ranges from 3,000 to 8,000 μg/L [42]. Several dogs in the present study had serum CRP values >8,000 μg/L, suggesting more severe joint inflammation and greater risk of disease progression [43]. We also found that synovial/serum CRP ratio in partial CR stifles was correlated with progressive CrCL fiber tearing, supporting the concept that CRP reflects severity and progression of synovitis/OA [44,45] and, additionally, non-contact complete CR in dogs.

High serum CXCL8 is associated with development of complete CR in dogs [34]. High serum CXCL8 was also found in several dogs at diagnosis in the present study. CXCL8 functions as a chemoattractant of neutrophils and macrophages to sites of inflammation and is a potent promoter of angiogenesis. Local expression of CXCL8 in CrCL tissue and stifle synovium is typical in stifles with CR [46]. Higher serum CCL2, GM-CSF, IFNγ, and IL-10 were also found at 8 weeks in dogs that developed a second CR. These observations suggest CR is associated with immune-regulatory events that include recruitment of monocyte macrophages to sites of inflammation and up-regulation of CXCL8. Although poorly understood, mechanical loading of ligament can influence expression of CCL2 and related cytokines, potentially linking the CrCL mechanical environment and development of local inflammatory events that influence CrCL homeostasis over time [47,48].

MSC treatment of chronic inflammatory diseases in human, such as chronic obstructive pulmonary disease, induces suppression of serum CRP [49]. In the present study, treatment with BM-MSCs was also associated with suppression of serum and to a lesser extent synovial CRP. Since BM-MSCs were not injected into the TPLO-treated stifle, where significant suppression of synovial CRP was found, this may suggest that synovial CRP is influenced by systemic injection of BM-MSCs, although it is also possible synovial CRP was influenced by the joint stabilizing effect of TPLO. BN analysis indicated that serum CRP and synovial CRP in the unstable CR stifle was influenced by BM-MSC injection, whereas this conditional dependency was weak in partial CR stifle synovial fluid.

BM-MSC injection induced few changes in T cell subsets within the peripheral circulation. However, reduction in numbers of circulating CD8^+^ T cells at 8 weeks was evident. BM-MSC injection effects on circulating T cell subsets was variable in individual dogs. Suppression of high initial counts was found in several dogs, while in other dogs, elevation in T cell subset numbers occurred after injection. In the mouse collagen arthritis model, treatment with MSCs reduces expression of pro-inflammatory cytokines in inflamed joints and serum, and increases expression of the anti-inflammatory cytokine IL-10 [50]. Further investigation of BM-MSC—CD8^+^ T cell interactions in dogs with CR is warranted.

BM-MSC injection also induced changes in serum and stifle synovial fluid cytokine concentrations. The increases in serum CXCL8 after BM-MSC injection were not associated with significant changes in synovial CXCL8, although higher synovial CXCL8 was found in the partial CR stifles after intra-articular injection of BM-MSCs. The correlation between serum CXCL8 and CRP became much weaker after BM-MSC injection. Changes in CXCL8 concentrations are likely a consequence of BM-MSC injection, as secretion of CXCL8 and related chemokines is a typical feature of cultured BM-MSCs [36]. In stifle synovial fluid, suppression of IFNγ and IL-6 was also evident after BM-MSC injection. These observations likely reflect the immune-modulatory effects of BM-MSCs on macrophages and T lymphocytes [49,51–54]. Correlation of synovial CCL2 with other cytokines in both stifles was enhanced after BM-MSC injection. Importantly, the pattern of correlation between synovial cytokines was strongly and positively enhanced by BM-MSC injection in the partial CR stifle, suggesting that the MSCs are acting on an upstream master regulator, such as a transcription factor, that influences the cytokine network within the stifle joint environment. At 8 weeks, stifle synovial/serum CRP ratio was higher than at diagnosis in dogs with partial CR that did not experience a second CR, suggesting that additional anti-inflammatory treatment may be needed to augment potential risk reduction second CR [49]. Enhancement of immunomodulation by MSCs could be accomplished in several ways [55–57]. For example, treatment with substance P during culturing of late passage cells could reduce senescence [55]. Use of a three-dimension spheroid culture method for MSCs enhances MSC production of prostaglandin E2 (PGE2) and anti-inflammatory actions on macrophages [56]. Finally, the tissue source used for preparation of cultured MSCs may influence immunomodulatory properties [57].

There were several limitations to our study. A relatively small cohort of client-owned dogs was used and histological analysis of synovium and ligament was consequently limited. Analysis of B lymphocytes and monocytes in the peripheral circulation and macrophages within the stifle joint may have aided understanding of BM-MSC interactions with the immune system. Analysis of additional cytokines, such as the major pro-inflammatory cytokine IL-1β [58], may have aided interpretation of our results, although limited volumes of synovial fluid were available for analysis. Variation in passage number is known to influence immune-modulatory effects of MSCs. In one individual dog, expansion of BM-MSC cells to the required dosage was slow and cells were transplanted at passage 5. Numbers of BM-MSCs used for intravenous and intra-articular injection was arbitrary, but similar to other studies [6,49]. This study was not powered to enable survival analysis of time to contralateral CR as an outcome measure [1]. Nevertheless, the results of the present study strongly demonstrate anti-inflammatory effects of BM-MSC injection and suggest that additional work should be pursued to optimize this promising therapeutic approach. There is a great need for a clinically effective disease-modifying therapy for cruciate ligament rupture in dogs with a low risk of adverse effects on ligament matrix [15]. In this regard, non-steroidal anti-inflammatory drug treatment, such as robenacoxib, also has the potential to modify joint inflammation and reduce synovial CRP in client-owned dogs with CR [59].

## Conclusions

This study is the first to investigate use of autologous BM-MSC injections as an anti-inflammatory treatment for CR in dogs. We have documented a suppressive effect of autologous BM-MSCs on serum and synovial CRP and related immune-modulatory effects on serum and stifle synovial fluid cytokines that is sustained over an 8-week period. Local intra-articular injection of BM-MSCs into the stable partial CR stifle was associated with profound alteration in the pattern of correlation of synovial fluid cytokines, suggesting MSCs influence an upstream regulator of related pathways. We also found that synovial/serum CRP ratio in partial CR stifles is a predictive biomarker for risk of a second CR. These results highlight the potential value in use of autologous BM-MSCs to suppress stifle inflammation in canine CR patients or human beings with ACL rupture that receive surgical treatment with an intra-articular graft.

## Supporting Information

S1 File Study data is provided in the supporting Excel file PONE-D-16-15516 data. (XLS)

S1 Table Absolute T lymphocyte subset numbers in the peripheral circulation before and after BM-MSC treatment. (DOCX)

S2 Table T lymphocyte subset proportions in the peripheral circulation before and after BM-MSC treatment. (DOCX)

S3 Table Association of systemic and local biomarkers with development of a second cruciate ligament rupture (CR) in a group of dogs with unilateral CR and contralateral stable partial CR at diagnosis. (DOCX)
