# Supplementary material for: Autologous Bone Marrow-Derived Mesenchymal Stem Cells Modulate Molecular Markers of Inflammation in Dogs with Cruciate Ligament Rupture
Source: PLoS One. 2016 Aug 30;11(8):e0159095. doi: 10.1371/journal.pone.0159095 (PMC5005014; doi:10.1371/journal.pone.0159095)
Supplement: S1 Table — (DOCX) [file pone.0159095.s002.docx]

**Table S1. Absolute T lymphocyte subset numbers in the peripheral circulation before and after BM-MSC treatment**

| **Epitope** | **Dog** | **Diagnosis** | **4 weeks** | **8 weeks** |
| --- | --- | --- | --- | --- |
| CD3^+^ | 1 | 0.227 |  | 0.054 |
|  | 2 | 0.055 |  |  |
|  | 3 | 0.047 |  | 0.112 |
|  | 4 | 0.135 | 0.014 | 0.044 |
|  | 5 | 0.001 | 0.000 | 0.000 |
|  | 6 | 0.029 | 0.004 | 0.145 |
|  | 7 | 0.013 | 0.042 | 0.019 |
|  | 8 | 0.070 | 0.008 | 0.092 |
|  | 9 | 0.009 | 0.898 | 0.172 |
|  | 10 | 0.357 |  | 0.037 |
|  | 11 | 0.006 | 0.005 | 0.009 |
|  | 12 | 0.107 | 0.006 | 0.013 |
| CD4^+^ | 1 | 0.094 |  | 0.027 |
|  | 2 | 0.019 |  |  |
|  | 3 | 0.027 |  | 0.064 |
|  | 4 | 0.061 | 0.009 | 0.026 |
|  | 5 | 0.001 | 0.000 | 0.000 |
|  | 6 | 0.013 | 0.002 | 0.07 |
|  | 7 | 0.003 | 0.025 | 0.012 |
|  | 8 | 0.035 | 0.004 | 0.052 |
|  | 9 | 0.005 | 0.614 | 0.114 |
|  | 10 | 0.171 |  | 0.018 |
|  | 11 | 0.004 | 0.003 | 0.006 |
|  | 12 | 0.075 | 0.004 | 0.009 |
| CD8^+^ | 1 | 0.091 |  | 0.016 |
|  | 2 | 0.009 |  |  |
|  | 3 | 0.007 |  | 0.020 |
|  | 4 | 0.019 | 0.003 | 0.008 |
|  | 5 | 0.000 | 0.000 | 0.000 |
|  | 6 | 0.014 | 0.002 | 0.058 |
|  | 7 | 0.009 | 0.008 | 0.004 |
|  | 8 | 0.017 | 0.002 | 0.024 |
|  | 9 | 0.002 | 0.189 | 0.032 |
|  | 10 | 0.110 |  | 0.008 |
|  | 11 | 0.001 | 0.001 | 0.001 |
|  | 12 | 0.013 | 0.001 | 0.002 |
| CD4^-^CD8^-^ | 1 | 0.099 |  | 0.023 |
|  | 2 | 0.048 |  |  |
|  | 3 | 0.030 |  | 0.056 |
|  | 4 | 0.090 | 0.006 | 0.021 |
|  | 5 | 0.001 | 0.000 | 0.000 |
|  | 6 | 0.009 | 0.002 | 0.061 |
|  | 7 | 0.004 | 0.017 | 0.008 |
|  | 8 | 0.032 | 0.005 | 0.031 |
|  | 9 | 0.005 | 0.378 | 0.069 |
|  | 10 | 0.095 |  | 0.047 |
|  | 11 | 0.002 | 0.004 | 0.005 |
|  | 12 | 0.038 | 0.001 | 0.007 |
| CD4^+^ CD8^+^ | 1 | 0.000 |  | 0.000 |
|  | 2 | 0.000 |  |  |
|  | 3 | 0.000 |  | 0.000 |
|  | 4 | 0.000 | 0.000 | 0.000 |
|  | 5 | 0.000 | 0.000 | 0.000 |
|  | 6 | 0.005 | 0.001 | 0.012 |
|  | 7 | 0.001 | 0.000 | 0.000 |
|  | 8 | 0.000 | 0.000 | 0.000 |
|  | 9 | 0.000 | 0.000 | 0.000 |
|  | 10 | 0.000 |  | 0.000 |
|  | 11 | 0.000 | 0.000 | 0.000 |
|  | 12 | 0.000 | 0.000 | 0.000 |

**Note**: Absolute counts (E06/ml of blood). BM-MSC – bone marrow-derived mesenchymal stem cells.
