# Supplementary material for: Autologous Bone Marrow-Derived Mesenchymal Stem Cells Modulate Molecular Markers of Inflammation in Dogs with Cruciate Ligament Rupture
Source: PLoS One. 2016 Aug 30;11(8):e0159095. doi: 10.1371/journal.pone.0159095 (PMC5005014; doi:10.1371/journal.pone.0159095)
Supplement: S2 Table — (DOCX) [file pone.0159095.s003.docx]

**Table S2. T lymphocyte subset proportions in the peripheral circulation before and after BM-MSC treatment**

| **Epitope** | **Dog** | **Diagnosis** | **4 weeks** | **8 weeks** |
| --- | --- | --- | --- | --- |
| CD3^+^ | 1 | 80 |  | 80 |
|  | 2 | 72 |  |  |
|  | 3 | 73 |  | 80 |
|  | 4 | 79 | 77 | 80 |
|  | 5 | 63 | 70 | 80 |
|  | 6 | 91 | 77 | 83 |
|  | 7 | 82 | 84 | 80 |
|  | 8 | 82 | 74 | 85 |
|  | 9 | 73 | 76 | 80 |
|  | 10 | 94 |  | 50 |
|  | 11 | 82 | 65 | 72 |
|  | 12 | 84 | 92 | 73 |
| CD4^+^ | 1 | 33 |  | 41 |
|  | 2 | 25 |  |  |
|  | 3 | 42 |  | 46 |
|  | 4 | 36 | 50 | 48 |
|  | 5 | 30 | 32 | 36 |
|  | 6 | 42 | 34 | 40 |
|  | 7 | 20 | 49 | 50 |
|  | 8 | 41 | 39 | 48 |
|  | 9 | 43 | 52 | 53 |
|  | 10 | 45 |  | 25 |
|  | 11 | 56 | 35 | 48 |
|  | 12 | 59 | 66 | 52 |
| CD8^+^ | 1 | 32 |  | 24 |
|  | 2 | 11 |  |  |
|  | 3 | 11 |  | 14 |
|  | 4 | 11 | 16 | 14 |
|  | 5 | 26 | 20 | 20 |
|  | 6 | 43 | 43 | 33 |
|  | 7 | 57 | 16 | 18 |
|  | 8 | 20 | 19 | 22 |
|  | 9 | 16 | 16 | 15 |
|  | 10 | 29 |  | 11 |
|  | 11 | 14 | 12 | 10 |
|  | 12 | 10 | 10 | 9 |
| CD4^-^CD8^-^ | 1 | 35 |  | 34 |
|  | 2 | 63 |  |  |
|  | 3 | 46 |  | 40 |
|  | 4 | 53 | 33 | 38 |
|  | 5 | 43 | 48 | 41 |
|  | 6 | 30 | 44 | 35 |
|  | 7 | 28 | 33 | 31 |
|  | 8 | 38 | 42 | 29 |
|  | 9 | 39 | 32 | 32 |
|  | 10 | 25 |  | 64 |
|  | 11 | 31 | 54 | 41 |
|  | 12 | 30 | 23 | 38 |
| CD4^+^ CD8^+^ | 1 | 0 |  | 0 |
|  | 2 | 0.56 |  |  |
|  | 3 | 0 |  | 0 |
|  | 4 | 0 | 0 | 0 |
|  | 5 | 0 | 0 | 0 |
|  | 6 | 14.5 | 14 | 7 |
|  | 7 | 7 | 0 | 0 |
|  | 8 | 0 | 0 | 0 |
|  | 9 | 0 | 0 | 0 |
|  | 10 | 0 |  | 0 |
|  | 11 | 0 | 0 | 0 |
|  | 12 | 0 | 0 | 0 |

**Note**: Proportional counts (% of peripheral blood mononuclear cells). BM-MSC – bone marrow-derived mesenchymal stem cells.
