# Supplementary material for: Autologous Bone Marrow-Derived Mesenchymal Stem Cells Modulate Molecular Markers of Inflammation in Dogs with Cruciate Ligament Rupture
Source: PLoS One. 2016 Aug 30;11(8):e0159095. doi: 10.1371/journal.pone.0159095 (PMC5005014; doi:10.1371/journal.pone.0159095)
Supplement: S3 Table — (DOCX) [file pone.0159095.s004.docx]

**Table S3**. Association of systemic and local biomarkers with development of a second cruciate ligament rupture (CR) in a group of dogs with unilateral CR and contralateral stable partial CR at diagnosis

| **Parameter** | **Dog #4** | **Dog #9** | **Dog #10** | **Dogs without a second CR (n = 9)** |
| --- | --- | --- | --- | --- |
| Radiographic synovial effusion score | 2 | 2 | 2 | 1 (1, 2) |
| Radiographic osteophyte score | 1 | 2 | 1 | 1 (1, 3) |
| Arthroscopy NRS score | 47 | 51 | 44 | 42 (32, 53) |
| Normalized CrCL_d_ | 0.151 | 0.156 | 0.168 | 0.157±0.008 |
| Peripheral blood CD4^+^ count | 0.061 | 0.005 | 0.171 | 0.019 (0.001, 0.094) |
| Peripheral blood CD8^+^ count | 0.019 | 0.002 | 0.110 | 0.009 (0.000, 0.091) |
| Peripheral blood CD4^-^CD8^-^ count | 0.090 | 0.005 | 0.095 | 0.03 (0.001, 0.099) |
| TNCC | 0.21 | 1.69 | 0.00 | 0.84 (0.00, 11.45) |
| Serum CRP | 4,134 | 1,313 | 17,495 | 3,168 (955, 65,688) |
| Synovial CRP | 912 | 337 | 3,968 | 429 (131, 9,815) |
| Synovial/Serum CRP ratio | 0.220 | 0.257 | 0.227 | 0.12 (0.089, 0.197) |
| Serum CCL2 | 112 | 60 | 137 | 225 (85, 831) |
| Serum IL18 | 0.0 | 5.7 | 0.0 | 12.4 (0, 39.1) |
| Synovial IFNγ | 7.7 | 0.0 | 14.7 | 28.3 (1, 83.8) |

**Note**: Synovial effusion severity score range was 0-2. Osteophyte severity score range was 0-3. Absolute T lymphocyte counts (E06/ml of blood). T cell identity was confirmed by CD3 staining. Synovial CRP and synovial/serum CRP ratio represent data for the stable partial CR stifle. CrCL_d_ was normalized to the length of the patella to account for variation in dog size. **Abbreviations**: TNCC, total nucleated cell count (x1000/μL); CRP, C-reactive protein (μg/L), CrCL, cranial cruciate ligament.
